# Nano-hydroxyapatite/polyaniline composite as an efficient sorbent for sensitive determination of the polycyclic aromatic hydrocarbons in air by a needle trap device†

**DOI:** 10.1039/d0ra07540j

**Published:** 2020-11-19

**Authors:** Ali Akbar Alinaghi Langari, Saber Alizadeh, Shiva Soury, Ali Firoozichahak, Davood Nematollahi, Parsa Mohammad Alizadeh, Nasim Sanaei

**Affiliations:** Student Research Committee, School of Public Health, Bam University of Medical Sciences Bam Iran; Department of Chemistry, Bu-Ali-Sina University Hamedan Iran; Department of Occupational Health Engineering, School of Public Health, Ilam University of Medical Sciences Ilam Iran; Department of Occupational Health, Faculty of Health, Social Determinants of Health Research Center, Gonabad University of Medical Sciences Gonabad Iran a.firoozi@edu.umsha.ac.ir; Center of Excellence for Occupational Health, Occupational Health and Safety Research Center, School of Public Health, Hamadan University of Medical Sciences Hamadan Iran

## Abstract

Hydroxyapatite is a readily available, inexpensive, environmentally friendly adsorbent with high adsorption capacity. In this study, a polyaniline-doped nano-hydroxyapatite (PANI@HA) adsorbent was synthesized and employed in a needle trap device for the extraction of polycyclic aromatic hydrocarbons such as naphthalene, fluoranthene, benzo[*a*]anthracene, phenanthrene, and benzo[*a*]pyrene for the first time. The synthesized adsorbent was characterized by X-ray diffraction, field emission scanning electron microscopy, and Fourier-transform infrared spectroscopy analysis. Initially, effective variables such as the carryover effect, storage time, accuracy, and precision of the method were examined in the laboratory. The desorption conditions were optimized using the response surface methodology and central composite design methods. From the standpoint of quantitative parameters, the limit of detection and limit of quantitation were determined to be between 0.001 and 0.003 and 0.021 and 0.051 ng mL^−1^, respectively, which indicates the high sensitivity of the proposed method. Additionally, no significant changes were detected after storage of analytes inside the needle at 4 °C after 60 days. The results of this study also provide a high correlation between the results of sampling with needles containing PANI@HA and with XAD-2 adsorbent tubes (standard NIOSH 5115 method) (*R*^2^ = 0.98). Finally, the proposed method was successfully employed in the extraction and determination of polycyclic aromatic hydrocarbons in field (real) samples. In general, it can be concluded that a needle packed with PANI@HA is a reliable and high-performance method for sampling polycyclic aromatic hydrocarbons compared to the NIOSH method.

## Abstract 2

Nano-HA/PANI was used in NTDs as a solvent less method and reuse several times.

## Graphical abstract

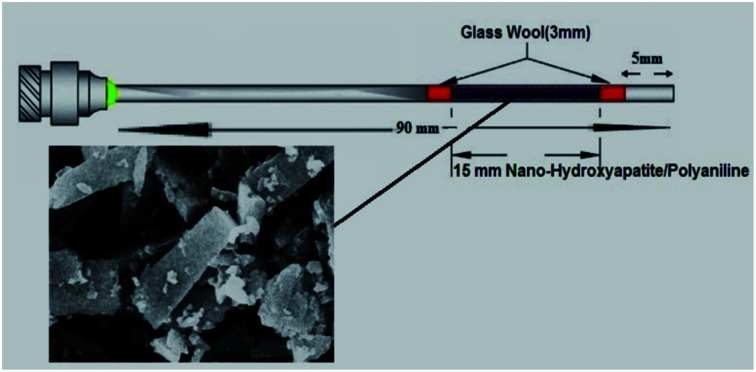

## 1. Introduction

Polycyclic aromatic hydrocarbons (PAHs) are a large group of organic compounds with at least two aromatic rings. These compounds can be obtained by incomplete pyrolysis of organic matter, which mainly originates from power plants, car engine outlets, cigarette smoke, and tobacco.^1^ To date, several PAHs, such as benzo[*a*]pyrene, have been classified as carcinogenic.^2–4^ OSHA has stated that the permissible limits for naphthalene, phenanthrene, and benzo[*a*]pyrene are 50.0, 0.2 and 0.2 mg m^−3^, respectively. Due to the carcinogenicity of these compounds, the introduction of a highly sensitive and efficient method for the determination of these pollutants in air is an important scientific goal. To date, different methods have been introduced for the sampling and analyzing of PAH compounds. NIOSH and OSHA are the recommended methods for the sampling of PAH compounds. In these methods, the extraction and preparation steps after the sampling process depend on the applied solvent. From the eco-friendly standpoint, large volumes of solvents are required for extraction, washing and recovery of the adsorbent, which can be expensive, toxic, carcinogenic, and hazardous to the environment and human health.^5,6^ In this regard, attention to solvent-free methods has increased significantly. Recently, the solid phase micro-extraction (SPME) and needle trap device (NTD) techniques as solvent-free methods have been widely considered by researchers.^7–9^ To date, various reports have been published on the SPME technique for the sampling and extraction of PAH compounds in different matrices.^1,10–13^ Despite the mentioned advantages, this method has some drawbacks, such as fiber fragility and limited fiber capacity. The needle trap device (NTD) was first introduced by Pawliszyn in 2001.^14^ Simple and rapid sampling of the pollutant in an unbalanced manner are the prominent features of the solvent-free NTD technique.^14,15^ Also, in the needle trap sampling method, more adsorbent can be placed inside the NTD, which increases the absorption capacity.^14,16^ The type of adsorbent employed in the NTD is very effective in determining its performance. Various commercial and synthetic adsorbents have been investigated in different studies.^17–20^ To the best of our knowledge, one efficient adsorbent that has not been used in NTDs is nano-hydroxyapatite particles (nHA). Hydroxyapatite (HA), with the chemical formula (Ca_10_(PO_4_)_6_(OH)_2_) and Ca/P = 1.67, is the main calcium phosphate material found in biological tissues such as vertebrae and teeth. Due to its chemical and structural similarity to bone mineral, it has been used in this sector in the medical field.^21^ The remarkable properties of nHA, such as high absorption capacity, biocompatibility, high stability in oxidation and reduction conditions, multiple interactions, low-cost preparation, non-toxicity, and high sensitivity, have attracted the attention of many scientists, including analytical chemists and environmental researchers.^22^ Compared to the other existing commercial adsorbents, nHA has provided new insight into sampling of analytes due to its small size, high surface area, low density, and high surface-to-volume ratio. Also, thermal stability is one of the unique features of this adsorbent; unlike metal–organic framework adsorbents,^23,24^ it can withstand high temperature conditions of up to 1450 °C.^25,26^ nHA and its derivatives are well-known as adsorbents for many compounds due to the presence of two C and P ionic bonds on their surfaces and the presence of cationic and phosphate ions. nHA has been used as a solid phase in high-efficiency liquid chromatography (HPLC),^27,28^ solid-phase extraction,^29–31^ chemical sensors and micro-solid phase extraction.^22,32,33^ However, researchers are attempting to optimize and enhance these remarkable abilities by building new HA nanoparticle composites. To strengthen the structural stability and increase the surface area of the adsorbents, a wide range of materials have been used. Recently, polyaniline (PANI) has been reported in studies.^34^ Because of its simple synthesis, high electrical conductivity, and long-term environmental stability, polyaniline is a potential candidate to be employed as a composite in the hydroxyapatite structure.^35^ The suitable interaction tendency of these materials is an important parameter.^36^ HA can have good chemical affinity with polyaniline. Moreover, interaction of polyaniline with nHA leads to increased mechanical strength, enhanced chemical stability and higher surface area.^37^ To the best of our knowledge, there is no study about the use of hydroxyapatite particles in NTDs for air pollution sampling. Additionally, polyaniline-doped nano-hydroxyapatite (PANI@HA) has not been used in any matrix for the sampling of PAHs. According to this background, in the present study, we attempted to use a PANI@HA composite adsorbent packed in a needle trap device for sampling and analyzing aromatic polyhydrocarbons (naphthalene, fluoranthene, benzo[*a*]anthracene, phenanthrene, and benzo[*a*]pyrene) by GC-FID. Optimization of the adsorption conditions (temperature and time) was performed using the statistical response methods of response surface methodology (RSM) and central composite design (CCD). In order to verify the performance of the NTD:PANI@HA method, analytical parameters such as the limit of detection (LOD), limit of quantification (LOQ), and linear dynamic range (LDR) were evaluated. Finally, the performance of NTD:PANI@HA was examined in real time.

## 2. Materials and methods

### 2.1 Chemicals and reagents

In this study, benzo[*a*]pyrene (99.2%), phenanthrene (99.8%), naphthalene (99.9%), fluoranthene (99.3%), benzo[*a*]anthracene (99.2%), calcium chloride (CaCl_2_) (98%), diammonium hydrogen phosphate ((NH_4_)_2_HPO_4_) (99%), potassium dihydrogen phosphate (KH_2_PO_4_) (98%), ammonium peroxydisulfate ((NH_4_)_2_S_2_O_8_) (99%), acetic acid (99.8%), hydrochloric acid (98%), sodium hydroxide (98%), and aniline (99%) were obtained from Sigma-Aldrich and used without any purification. Also, high purity nitrogen (99.99%) was obtained from Raham Company (Tehran, Iran).

### 2.2 Instruments

In this study, we used a 22-gauge (G) spinal needle (outer diameter 0.71 mm and a length of 90 mm) obtained from Kosan (Tokyo, Japan). A sampling pump (SKC 222 series, PA, USA) with a flow rate of 1–200 mL min^−1^ was connected to the NTD packed with PANI@HA. The XAD-2 adsorbent tubes (100 mg/50 mg) were obtained from the SKC for the sampling of the PAH compounds according to the NIOSH 5515 standard method. A magnetic hot plate (TAT-94-1062) (Tehran-Iran) was employed to heat the samples during the extraction process. Also, a 3.0 mL Luer-Lok syringe (Mina Tajhiz-Tehran) was applied to inject the carrier gas (nitrogen). A balancing scale (model BP-121s, Germany) was employed to weigh the analytes. A three-mouth flask was used to obtain certain concentrations of the analytes. Additionally, a digital thermometer (Testoterm GmbH) was set up for continuous monitoring of the inside temperature of the glass container. To investigate the crystal structure and purity of synthesized PANI@HA, the X-ray diffraction technique was performed with a Rigaku Ultima device made in Japan in transmission mode using Cu Kα radiation at 2*θ* = 5–80. A field-emission scanning electron microscope (FE-SEM, TESCAN Mira, Czech Republic) was employed for the morphology investigation. To confirm the functional groups in the synthesized structure of the adsorbent, a Fourier-transform infrared (FT-IR) spectrometer (Perkin Elmer, USA) was used.

### 2.3 Gas chromatography specifications

A Varian CP-3800 gas chromatograph with an FID detector equipped with a CP7462 column (30 m × 0.25 mm) was used for the separation of the PAH compounds. To study the thermal desorption of the PAH compounds, the temperature of the injection site was adjusted in the range of 270–390 °C. The GC was set up with a split-less liner, column flow of 1.6 mL min^−1^, and injection port pressure of 8.85 psi. Moreover, high purity nitrogen was used as a carrier gas at a flow rate of 1.6 mL min^−1^. The oven temperature program started at 80 °C and then reached 160 °C with a rate of 25 °C per minute. The temperature was then increased to 300 °C with a rate of 30 °C per minute and maintained at this temperature for 2 min. Finally, the temperature of the detector was set to 325 °C.

### 2.4 Response surface methodology

Response surface methodology (RSM) is a set of mathematical methods that determines the relationships between one or more response variables and several independent variables. Box and Wilson introduced this method in 1951. It is still used as a test design tool. In designing experiments, the goal is to identify and analysis the variables affecting the outputs with the fewest experiments. The response level method is a mathematical–statistical method for optimizing test outputs. In this study, the number of experiments was determined using response surface methodology (RSM) and central composite design (CCD). The software estimates the optimal point using eqn (1), in which *Y* represents the model prediction, *B*_0_ is the constant-coefficient, *B*_*i*_ is the linear coefficient, *B*_*ij*_ is the interaction coefficient between the variables, *X* is the variable and *ε* is the residual amount.^38^1
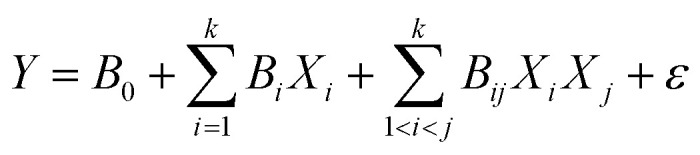


### 2.5 Adsorbent synthesis procedure

#### 2.5.1 nHA synthesis procedure

The employed nano-hydroxyapatite (nHA) in this study was synthesized by the reaction of CaCl_2_ with (NH_4_)_2_HPO_4_ based on previously reported studies.^35^ Briefly, 100 mL of (NH_4_)_2_HPO_4_ solution (0.4 M) was stirred at room temperature, and then 100 mL of CaCl_2_ solution (0.6 M) was added dropwise to produce a white precipitate. The pH of the reaction medium was adjusted and maintained at 10.0–11.0 by adding NaOH (0.1 N) solution during the reaction period. The obtained powder was rinsed three times with distilled water for removal of the remaining salts. The resulting precipitate was aged at temperatures of 24–90 °C for 24 hours and stored at room temperature.

#### 2.5.2 PANI synthesis procedure

Polyaniline (PANI) was prepared according to previous studies.^39^ First, 20.4 g of freshly distilled aniline monomer was dissolved in 230.0 mL HCl (1.5 M), and the resulting solution was cooled to below 5 °C for 1 hour. Also, 25.0 g of ammonium peroxydisulfate was dissolved in 250 mL of HCl (1.5 M). The pre-cooled solution was slowly added to the second solution. The reaction temperature was maintained at 0–5 °C for 4 h. The obtained dark green precipitate was thoroughly washed with distilled water. PANI was obtained as a dark green powder after drying at 40 °C for 48 hours.

#### 2.5.3 PANI@HA synthesis procedure

For the preparation of the PANI@HA composite, 5.0 g of synthesized polyaniline was added to 100.0 mL of ethanol in a three-neck open flask with a temperature of 70 °C. After complete dissolution of polyaniline, 2.9 g of synthesized nHA slurry (75.0 wt% nHA) was added to the solution. The mixture was heated and stirred for 2 hours at 70 °C. When the reaction completed, the resulting mixture was aged at room temperature for 24 hours and then thoroughly washed with deionized water and ethanol. The obtained PANI@HA composite was placed in a vacuum oven at 70 °C for 48 hours. By changing the weight ratio of nHA slurry, the nHA ratio in the final composite could be changed.^40,41^

### 2.6 Sampling chamber

**Fig. 1:**
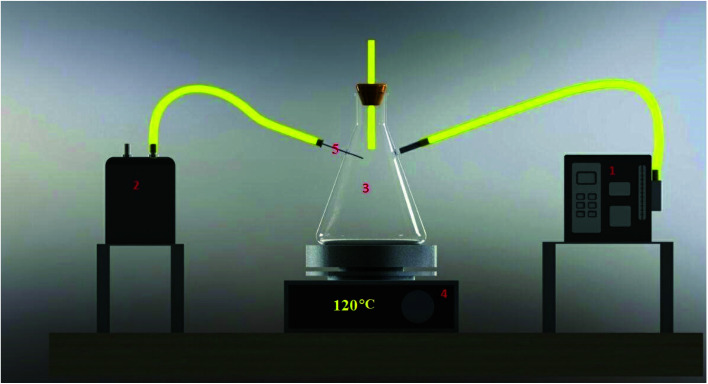
The scheme of the sampling chamber: (1) sampling pump (SKC Universal 44XR, USA) by the NIOSH 5515 method. (2) Sampling pump (SKC 222-3, USA) by the NTD:PANI@HA method. (3) A three-mouth flask. (4) Magnetic heater. (5) The needle trap device (gauge 22).

The sampling chamber is shown in Fig. 1. In this study, a three-mouth flask (250 mL) was used for the sampling of PAH compounds. First, for the preparation of different concentrations of analytes, all three-glass flask openings were closed. Amounts of 0.5–10.0 mg of the analytes were placed in the chamber at different times. Sampling began after the container was placed on the heater at 120 °C for 30 minutes. One of the paths in the glass flask was intended to allow entrance of fresh air and prevent vacuuming. The second path was used for the sampling of PAH compounds by the XAD-2 adsorbent tube (according to the NIOSH 5515 method). Finally, the third path was employed for the sampling of the analytes by the proposed NTD method. It should be noted that the sampling process was performed using the NIOSH and NTD methods simultaneously.

### 2.7 Preparation of NTD

In this study, gauge 22 medical spinal needles (O.D. 0.71 mm, I.D. 0.39 mm) were employed to fabricate the NTDs. The needle was packed with 1.0 mg of PANI@HA adsorbent and 1.0 mg of crushed glass particles (to prevent NTD blockage). Preliminary tests showed that no absorption occurred on the crushed glass. Both sides of the adsorbent inside the needle were closed with 3.0 mm glass wool, and a free gap of 5.0 mm was considered after the second layer of glass wool. The packed NTDs were connected to a low-flow sampling pump, and the flow rate was measured using a soap bubble flow meter. The optimum flow rate of 3.0 mL per minute was selected for sampling by the NTD method.

### 2.8 Desorption parameters

In this study, the desorption temperature was investigated in the range of 270–390 °C. The minimum temperature that creates the maximum peak area was selected as the optimal temperature for subsequent analyses. Also, the NTD was placed into the injection section of the GC for 3 to 12 minutes to measure the optimal desorption time. The temperature and desorption time were optimized using Design-Expert software version 10. The NTD with a Luer-Lok syringe was inserted into the injection site according to the specified desorption time range (3–12 minutes). After the desorption time, the carrier gas (nitrogen) was passed through the analytes and entered the GC column for detection of the values.

### 2.9 Method validation

The LOD, LOQ, and LDR parameters were assessed for validation of the NTD performance. To obtain the LOD and LOQ, a diluent was employed to reduce the known concentration of the analyte. The diluent was injected into the device each time to reach the desired concentrations at which the height of the obtained peak from the analysis was 3 and 10 times the background peak, respectively. Also, the LDR, which is an important analytical performance parameter, was investigated. To determine the accuracy of the results obtained by the NTD, side-by-side sampling was performed using the NTD and the standard method for each combination. In this section, the NIOSH 5515 method was used for sampling of the mentioned compounds. The accuracy of the concentration range was 0.5 to 2 times the allowable exposure limit.

### 2.10 Carryover measurement

After sampling, to determine the carryover value, the heating of absorption was performed in the injection section of the GC device at the specified times (3–12 minutes) again. The obtained peak ratios (of the second absorption to the first absorption) were expressed in terms of percentage to calculate the carryover values at the different times.

### 2.11 Determining precision

The precision of the measurements was calculated to determine the repeatability and reproducibility of the proposed method. To express the precision, the relative standard deviation index (RSD) was calculated. To calculate the repeatability, sampling was performed with an NTD at different concentration levels (5 different concentrations). The reproducibility was determined by sampling with three similar NTDs at one concentration.

### 2.12 Breakthrough volume

The breakthrough volume (BTV) is an important factor in preventing gross measurement errors. For this purpose, two NTDs packed with equal amounts of the studied adsorbent were connected by a linker tube. After the sampling process, the second NTD was injected into the GC, and the remaining analytes on the adsorbent bed were examined.

### 2.13 Determination of storage stability

For the determination of storage stability, certain concentrations of the mentioned analytes (naphthalene at a concentration of 50.0 mg m^−3^; phenanthrene, benzo[*a*]pyrene, fluoranthene and benzo[*a*]anthracene at a concentration of 0.2 ng mL^−1^) were sampled by the NTD under the optimal sampling conditions. The analysis process was performed over 1 to 60 days at laboratory (25 °C) and refrigerator (4 °C) temperatures. To store the samples, the NTD was covered with a septum and then stored for a specified period.

### 2.14 Field measurement

For evaluation of the proposed NTD in real conditions, 10 samples of regional air with high traffic and congestion of gasoline vehicles were sampled by NTD:PANI@HA. Finally, the results obtained with the XAD-2 adsorption tube were compared with the collected data from the NTD packed with PANI@HA.

## 3. Results and discussion

### 3.1 Characterization of the PANI@HA composite

**Fig. 2:**
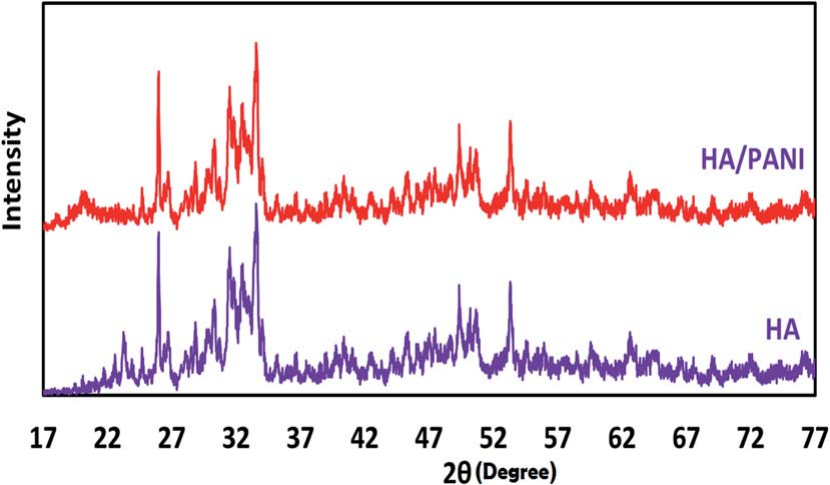
PXRD patterns of the nHA and PANI@HA structures.

The X-ray diffraction pattern presented in Fig. 2 is consistent with the previously reported data and shows the suitable crystallinity and successful synthesis of the PANI@HA adsorbent. The distinguished diffraction peaks can be assigned as hexagonal phase, consistent with the standard pattern of HA.^34,36^ The XRD pattern of nHA indicates the existence of all characteristic peaks at 2*θ* = 25.99, 31.77, 33.18, 35.16, 49.43 and 54.66°. These results prove the purity and suitable crystallinity of the synthesized nHA. In other words, calcium oxide and tri-calcium phosphate were not detected. These results are consistent with previously reported studies.^34,36^ However, the broad shoulder at 2*θ* = 20.44° corresponding to PANI cannot be seen clearly in the XRD patterns of the composites because the diffraction peaks of PANI are weak and are covered by the background of the HA peaks.^36^

**Fig. 3:**
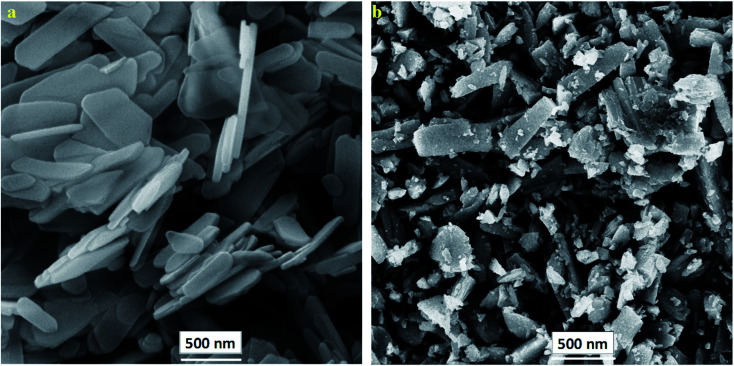
FE-SEM images of (a) nHA and (b) PANI@HA structures.

To investigate the synthesis and morphology of the prepared PANI@HA composites, FE-SEM analysis was performed. According to Fig. 3(a), the synthesized HA consists of unique flake-like structures. Fig. 3(b) indicates that the flake-like HA structures are doped with spherical agglomerates of PANI particles.

**Fig. 4:**
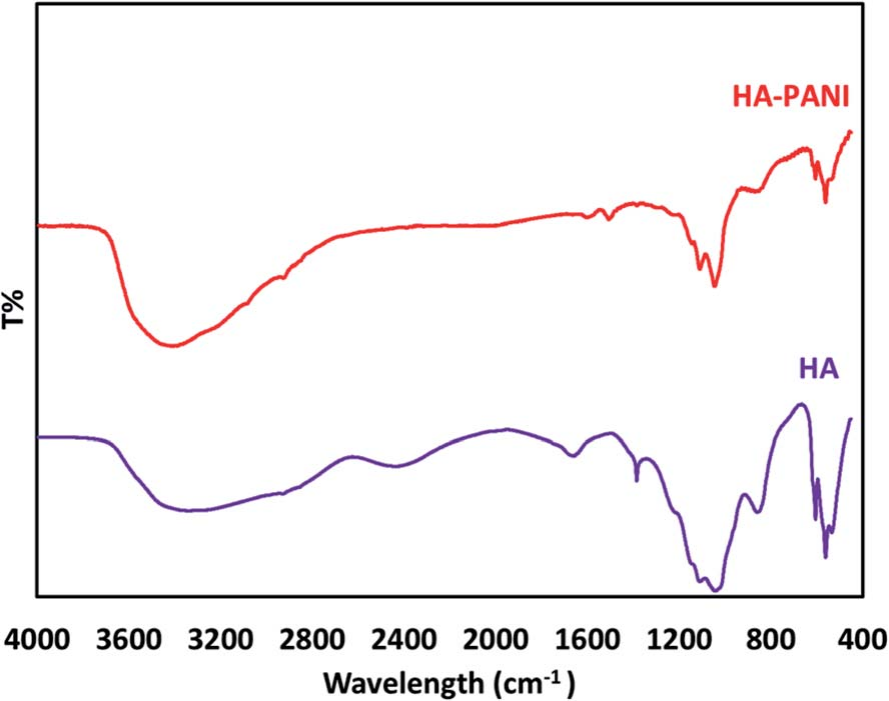
The FT-IR spectra of the HA and PANI@HA structures.

To confirm the formation of functional groups in the PANI@HA structure, the FT-IR spectra of HA and the PANI@HA composite were recorded. Fig. 4 indicates the FT-IR spectra of pure HA and the PANI@HA structures. The FT-IR spectrum of HA shows the specific peaks corresponding to the phosphate bending vibration mode (567, 603 cm^−1^), phosphate stretching vibration mode (963 cm^−1^), and phosphate stretching vibration mode (1035, 1101 cm^−1^). The FT-IR spectrum of the PANI@HA composite shows the specific bands of HA and PANI. The characteristic bands at 1301 and 1147 cm^−1^ can be assigned to the stretching mode of C–N in Ar–N (1301 cm^−1^), respectively. However, the intensity of the PANI peaks is relatively weak due to the low mass content and interaction with the composite.^37,42^

### 3.2 Desorption parameters

The desorption process is one of the most important steps in the analysis of trapped analytes in an NTD system. The effects of the desorption time and the temperature were modelled and optimized by Design-Expert software using response surface methodology (RSM) and central composite design (CCD). In this study, the desorption time and temperature were investigated between 3 and 12 minutes and 270 °C and 390 °C, respectively. In previously reported studies, micro-extraction and NTD methods were used to select the desorption temperature range for the analysis of PAH compounds.^43,44^ For example, Soury *et al.* reported a zinc-based metal–organic framework as a sorbent in the NTD method for the extraction of polycyclic aromatic hydrocarbons in air with a desorption time and temperature range of 331–379 °C and 7–9 minutes, respectively.^45^ Also, Xiang-Feng *et al.* reported a MIL-53(Al) coating as a sorbent in the SPME method for the determination of polycyclic aromatic hydrocarbons by gas chromatography with a desorption time of 4 min.^46^ In one study, they used a UiO-66 coating as a sorbent in the SPME method for the determination of polycyclic aromatic hydrocarbons, with a reported temperature and desorption time of 290 °C and 10 minutes, respectively.^43^ In this work, the optimal desorption temperatures for benzo[*a*]pyrene, naphthalene, phenanthrene, fluoranthene and benzo[*a*]anthracene are 384 °C, 356 °C, 362 °C, 368 °C and 375 °C, respectively. Also, the optimal desorption times are 9.12, 9.33, 9.47, 9.46 and 9.55 min, respectively, which are consistent with similar studies.^43–46^

**Fig. 5:**
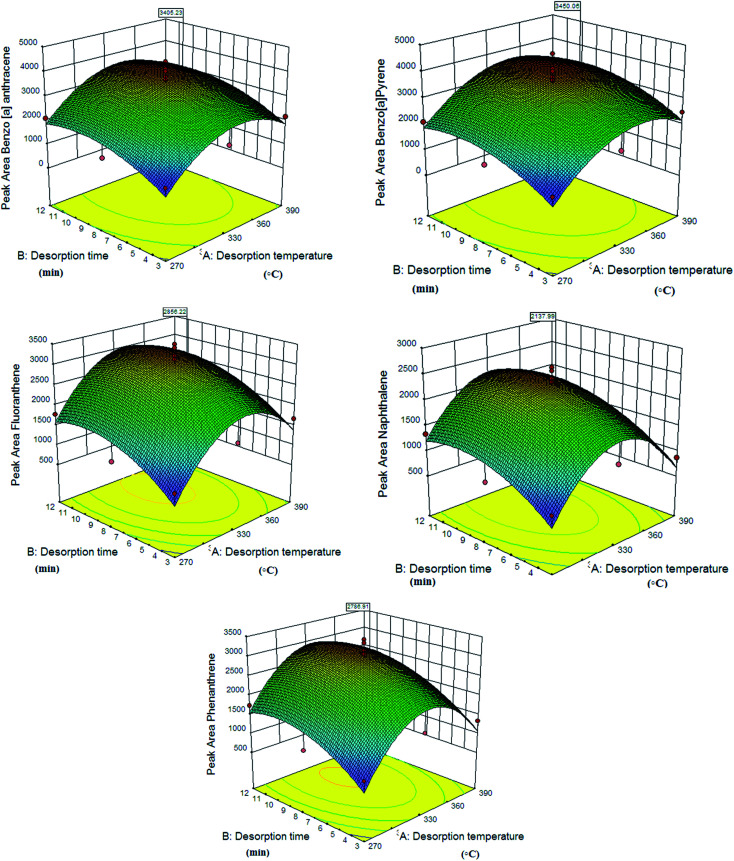
Effects of desorption variables on the efficiency of NTD packed with PANI@HA in the sampling and analysis of PAH compounds.

The obtained optimal desorption time for the analysis is higher than in other studies that used the solid phase micro-extraction method.^11,46^ This result can be related to the difference in the structures of SPME and NTD. In the SPME method, the adsorbed analyte can be separated from the adsorbent surface in a shorter time due to the direct contact of the adsorbent with the hot carrier gas. The predicted modelling data and optimal time and temperature values by RSM are given in Table 1. The calculated *R*^2^ values in this model are 82–84, indicating the appropriate response of the model. Fig. 5 shows the interactive effect of the desorption time and temperature on the NTD performance. Also, the chromatogram of the PAH compounds is shown in Fig. S1.†

**Table:** Analysis of variance (ANOVA) results for the desorption parameters

Parameters/analytes	Phenanthrene	Benzo[*a*]pyrene	Naphthalene	Fluoranthene	Benzo[*a*]anthracene
Temperature (°C)	362	384	356	368	375
Time (min)	9.47	9.12	9.33	9.46	9.55
*R*-Squared	0.84	0.84	0.86	0.83	0.82
Adj *R*-squared	0.73	0.72	0.76	0.71	0.70
SD	457.60	533.78	345.26	466.75	542.43
CV	20.84	18.86	20.92	20.22	19.47
Press	8.774 × 10^6^	1.014 × 10^3^	4.984 × 10^6^	9.129 × 10^6^	1.224 × 10^7^
Lack of fit	0.049	0.043	0.0535	0.049	0.052

### 3.3 Investigation of the breakthrough volume

The breakthrough volume (BTV) phenomenon can occur when the amount of trapped analytes in the back of the NTD is greater by 10% than the amount of trapped analytes in the front of the NTD. The sampling time was increased to 8 hours with increasing of the sampled air volume because the NTD airflow is constant (3.0 mL min^−1^). Based on these results, BTV was not seen even after 8 hours of sampling with the proposed NTD.

### 3.4 Carryover effect

The carryover effect depends on two parameters: desorption time and temperature. Soury *et al.* did not observe any carryover effect for the sampling of PAHs by the Zn-MOF adsorbent at the desorption time and temperature of 9 min and 379 °C, respectively.^45^ Moreover, in a similar study, the carryover effect was not observed for the sampling of polycyclic aromatic hydrocarbons by the MIL-53(Al) coating in the SPME method with a desorption time of 4 min.^46^ In this study, carryover was not observed at the optimal desorption time and temperature. However, the carryover effect was determined for the performed experiments with different desorption times and temperatures. These results are shown in Table 2. As can be seen in Table 2, the maximum carryover effect is related to the desorption temperature and time of 270 °C and 3 minutes, respectively.

**Table:** The carryover effect of the NTD packed with PANI@HA adsorbent at different desorption times and temperatures^a^

Temp. (°C)	Time (min)	Carryover effect for each analyte (%)				
		Naphthalene	Phenanthrene	Fluoranthene	Benzo[*a*]anthracene	Benzo[*a*]pyrene
330	7.5	0.12	0.15	0.16	0.18	0.2
330	12	0.10	0.10	0.11	0.11	0.12
270	12	0.14	0.15	0.15	0.16	0.17
330	3	0.21	0.22	0.27	0.29	0.33
390	7.5	ND	ND	ND	ND	ND
390	3	0.12	0.12	0.14	0.18	0.22
270	7.5	0.18	0.19	0.19	0.20	0.21
270	3	0.33	0.34	0.35	0.35	0.46
390	12	ND	ND	ND	ND	ND

a ND: not detected.

### 3.5 Method precision

To evaluate the precision, repeatability and reproducibility parameters, they were determined in terms of the RSD percentage. The repeatability for naphthalene at concentrations of 5.0, 10.0, 30.0, 50.0, and 100.0 mg m^−3^ and for phenanthrene, benzo[*a*]pyrene, fluoranthene and benzo[*a*]anthracene at concentration levels of 0.01, 0.02, 0.05, 0.07 and 1.0 mg m^−3^ was examined. The obtained results are shown in Table 3. The calculated RSD for the proposed NTD is between 2.8–9.9, which indicates appropriate precision of the sampling and analysis system in this study.

Relative standard deviation percentages for investigation of the reproducibility and repeatability of NTD:PANI@HA for extraction of PAH compounds

**Table:** 

Analytes	RSD% for a NTD at different concentrations (mg m^−3^)					RSD% for different NTDs at a constant concentration		
	5	10	30	50	100	NTD1	NTD2	NTD3
Naphthalene	4.3	5.8	9.1	9.9	8.6	4.5	6.4	10.5

**Table:** 

Analytes	RSD% for a NTD at different concentrations (mg m^−3^)					RSD% for different NTDs at a constant concentration		
	0.01	0.02	0.05	0.07	1	NTD1	NTD2	NTD3
Phenanthrene	3.9	4.5	3.2	5.8	4.4	10.7	4.8	3.8
Benzo[*a*]pyrene	2.8	3.9	5.1	4.3	4.9	5.5	11.3	5.9
Fluoranthene	3.2	2.7	4.8	4.1	4.3	8.8	10.1	8.5
Benzo[*a*]anthracene	2.9	3.3	4.1	5.6	3.4	9.4	7.6	4.7

To evaluate the reproducibility of the proposed method, three different NTDs with the same concentration (50.0 mg m^−3^ for naphthalene and 0.2 mg m^−3^ for phenanthrene, benzo[*a*]pyrene, fluoranthene and benzo[*a*]anthracene) were used in the optimal desorption conditions (Table 3). The precision of the samples was also evaluated at an acceptable level, which indicates appropriate reproducibility of the NTDs. It should be noted that there are no data about the repeatability and reproducibility of the NIOSH 5515 method for sampling of PAHs.^47^

### 3.6 Storage time

**Fig. 6:**
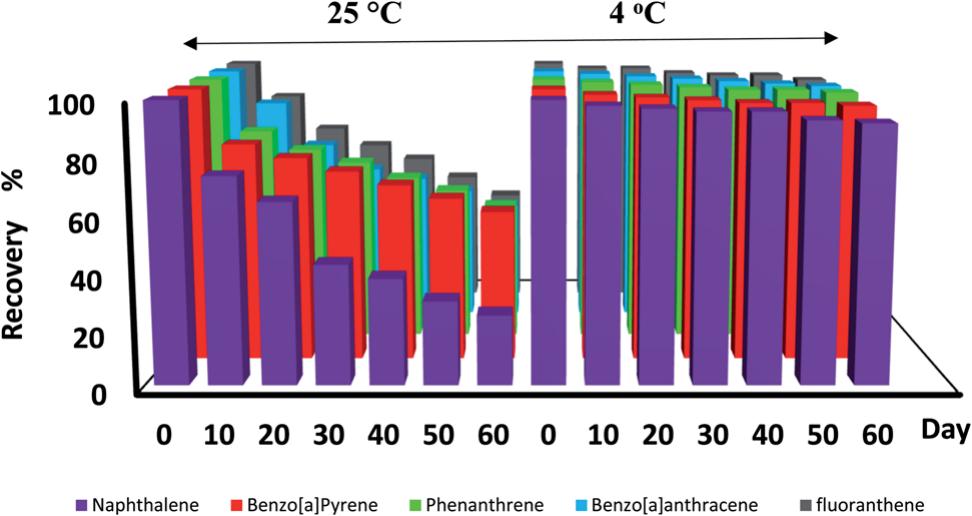
Storage times of PAH compounds trapped by NTD:PANI@HA from 1 day to 60 days at 4 °C and 25 °C.

Fig. 6 indicates the results of the storage time experiment. As shown in the diagram, the concentrations of the analytes (especially naphthalene) decreased below 70% at 25 °C after 20 days. On the other hand, there was no significant change in the peak surface area of the studied analytes at a temperature of 4 °C after 60 days. Therefore, the desired analytes can be stored in the proposed NTD for up to 60 days at refrigerator temperature. According to the NIOSH method for polyaromatic hydrocarbons, the sample should be protected from ultraviolet and heat radiation; however, the storage time is not specified in this method.^47^ Also, in the EPA TO-13A technique, the samples can be stored at 4 °C for 7 days.^48^

### 3.7 Validation of the method

The LOD and LOQ parameters for the NTD method were determined based on the signal-to-noise ratios of 3 to 1 and 10 to 1, respectively. The obtained results are in the range of 001–003 and 0.021–0.051 ng mL^−1^, respectively. The reported LOD by the NIOSH method (5515) for the analysis of PAH compounds is in the range of 0.3–0.5 μg per sample. The LOD value in this study is lower than that of the standard NIOSH method.^47^ Therefore, the suggested NTD offers high sensitivity for the determination of PAH compounds.

The LDR as another examined parameter for the NTD:PANI@HA method showed a linear range of up to 262.0 ng mL^−1^, which expresses the high adsorption capacity of the desired adsorbent. According to Table 4, the proposed NTD method shows a lower LOD and LOQ and higher linear range. Therefore, NTD:PANI@HA can be used to determine the concentrations of PAHs in a wide range in workplace air. Also, the accuracy parameters were investigated by comparison of the results obtained using the NTD:PANI@HA and XAD-2 methods at different concentrations for benzo[*a*]pyrene (Fig. S2†). The calculated *R*^2^ values in the range of 0.98–0.99 proved a good correlation coefficient between the two methods.

**Table:** Comparison of NTD:PANI@HA with other methods for sampling and analysis of PAH compounds^a^

Compounds	Current method (ng mL^−1^)			NTD/Zn-MOF GC/air (mg m^−3^)^45^			SPME/PDMS GC-MS/air (ng)^49^			MSPE/ZIF-7/GC-MS air–water (ng L^−1^)^50^		
	LOD	LOQ	LDR	LOD	LOQ	LDR	LOD	LOQ	LDR	LOD	LOQ	LDR
Naphthalene	0.002	0.021	0.002–398	0.011	0.04	0.01–262	0.02	0.05	NR	NR	NR	NR
Phenanthrene	0.001	0.051	0.001–8	0.021	0.07	0.021–1	0.92	3.06	NR	5.79	19.2	0.05–5
Benzo[*a*]pyrene	0.002	0.022	0.002–2	0.01	0.03	0.01–0.5	0.75	3.46	NR	3.32	11.1	0.05–5
Fluoranthene	0.003	0.031	0.003–3	NR	NR	NR	0.4	1.34	NR	5.38	17.9	0.05–5
Benzo[*a*]anthracene	0.002	0.024	0.002–3	NR	NR	NR	0.3	1.01	NR	NR	NR	NR

a NR: not reported.

### 3.8 Field measurements

After the employment of the NTD:PANI@HA method in laboratory-scale studies, the efficiency and performance of the method were examined in real conditions. To achieve this goal, samples of PAH compounds from regional air with high traffic and congestion of gasoline vehicles were evaluated, and the obtained results were compared with the NIOSH 5515 method (Table 5). It should be noted that in the NTD method, all of the adsorbed analytes were injected into the GC without dilution because of the solvent-free conditions. Therefore, the sensitivity of this method is higher than those of the usual methods.^51–56^

**Table:** The amounts of PAH compounds in air from a high-traffic area using the NTD:PANI@HA and NIOSH 5515 methods

Analyte	NTD:PANI@HA		NIOSH 5515:XAD2	
	Concentration (mg m^−3^)	RSD	Concentration (mg m^−3^)	RSD
Naphthalene	11.3	11.2	9.1	12.5
Phenanthrene	0.2	8.7	0.13	6.4
Benzo[*a*]pyrene	0.13	6.9	0.09	8.8
Fluoranthene	0.14	5.8	0.09	7.6
Benzo[*a*]anthracene	0.12	7.3	0.08	5.9

## 4. Conclusion

In this study, we attempted to provide NTD:PANI@HA as a promising method for the sensitive and efficient sampling and analyzing of PAH compounds for the first time. To achieve this goal, polyaniline-doped nano-hydroxyapatite as an efficient absorbent was synthesized and characterized by the PXRD, FE-SEM, and FT-IR techniques. Optimization of the adsorbent parameters was performed using the RSM and CCD methods. On the bench scale, studies of sampling and analysis parameters such as carryover effect, storage time, accuracy, and precision of the method presented suitable results. Additionally, the NTD packed with polyaniline-doped nano-hydroxyapatite was employed on the field scale to assess occupational and environmental exposure of PAHs. According to the obtained results, NTD:PANI@HA has a good adsorption capacity and high linear range. Comparison of the NTD technique with the NIOSH standard method proved that the proposed method is more suitable for sampling and analysis of PAH compounds in air and with a higher repeatability and sensitivity. The main problem with using NTD as a sampler is the absorption loading, which can lead to a drop in pressure and increase the sampling time. To address this drawback, it is recommended to use different adsorbent/crumb glass ratios in the preliminary tests and consider the best ratio for the main tests.

## Conflicts of interest

There are no conflicts to declare.

## Supplementary Material

RA-010-D0RA07540J-s001
