# Supplementary material for: Nano-hydroxyapatite/polyaniline composite as an efficient sorbent for sensitive determination of the polycyclic aromatic hydrocarbons in air by a needle trap device
Source: RSC Adv. 2020 Nov 19;10(69):42267–76. doi: 10.1039/d0ra07540j (PMC9057830; doi:10.1039/d0ra07540j)
Supplement: RA-010-D0RA07540J-s001 [file RA-010-D0RA07540J-s001.pdf]

Electronic Supplementary Material (ESI) for RSC Advances.
This journal is © The Royal Society of Chemistry 2020

Supporting Information

Nano-Hydroxyapatite/Polyaniline Composite as an Efficient Sorbent for Sensitive Determination of the Polycyclic Aromatic Hydrocarbons in the Air by the Needle Trap Device

Ali Akbar Alinaghi Langari[1], Saber Alizadeh [2], Shiva Soury[3], Ali Firoozichahak [4*], Davood Nematollahi [2], Parsa Mohammad Alizadeh[1], Nasim Sanaei[5]

[1] Student Research Committee, school of public health, Bam University of medical sciences, Bam, Iran

[2] Department of Chemistry, Bu-Ali-Sina University, Hamedan, Iran.

[3]Department of Occupational Health Engineering, School of Public Health, Ilam University of Medical Sciences, Ilam, Iran

[4] Department of Occupational Health, Faculty of Health, Social Determinants of Health Research Center, Gonabad University of Medical Science, Gonabad, Iran.

[5]Center of Excellence for Occupational Health, Occupational Health and Safety Research Center, School of Public Health, Hamadan University of Medical Sciences, Hamadan, Iran

Table S1: List of the abbreviations

SPME	Solid Phase Microextraction
NTD	Needle Trap Device
PAHs	Polycyclic Aromatic Hydrocarbons
LOD	Limit of Detection
LOQ	Limit of Quantitation
RSD	Relative Standard Deviation
BTV	Breakthrough Volume
FE-SEM	Field-Emission Scanning Electron Microscopy
XRD	X-ray Diffraction
FT-IR	Fourier-Transform Infrared Spectroscopy
Nano-HA/PANI	Nano-Hydroxyapatite/Poly-Aniline

Table S2: List of the Units

Parameters	Unit of Measure
Desorption Time	°C
Desorption Temperature	min
Breakthrough Volume	mL
Carryover Effect	Percentage (%)
Repeatability	Relative Standard Deviation Percentage
Reproduction	Relative Standard Deviation Percentage
Limit of Detection	$ng.mL^{-1}$
Limit of Quantitation	$ng.mL^{-1}$
Concentration	$mg.m^{-3}$

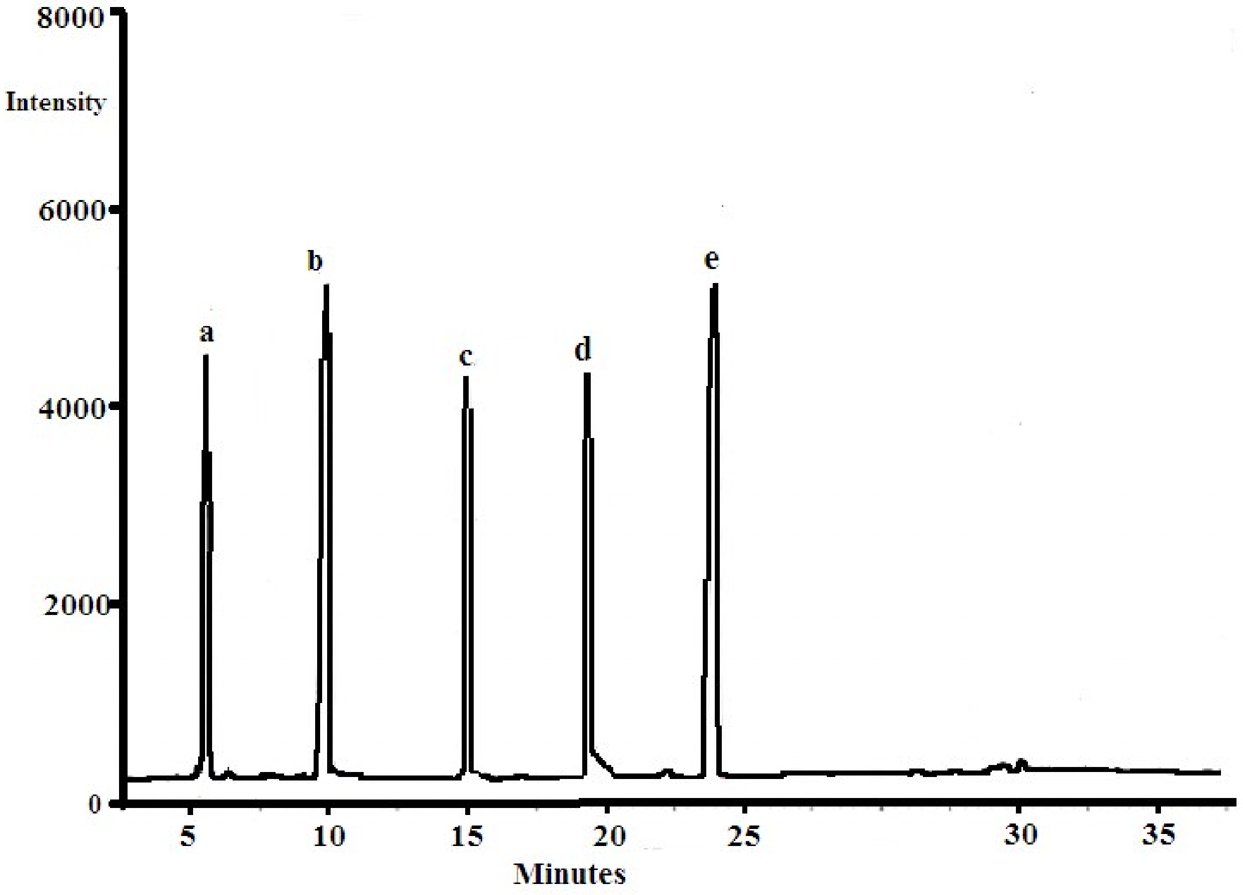

Figure S1. Chromatogram of PAHs compounds from NTD: PANI@HA. a) Naphthalene, b) Phenanthrene, c) Fluoranthene, d) Benzo[a]anthracene, and e) Benzo[a]pyrene.

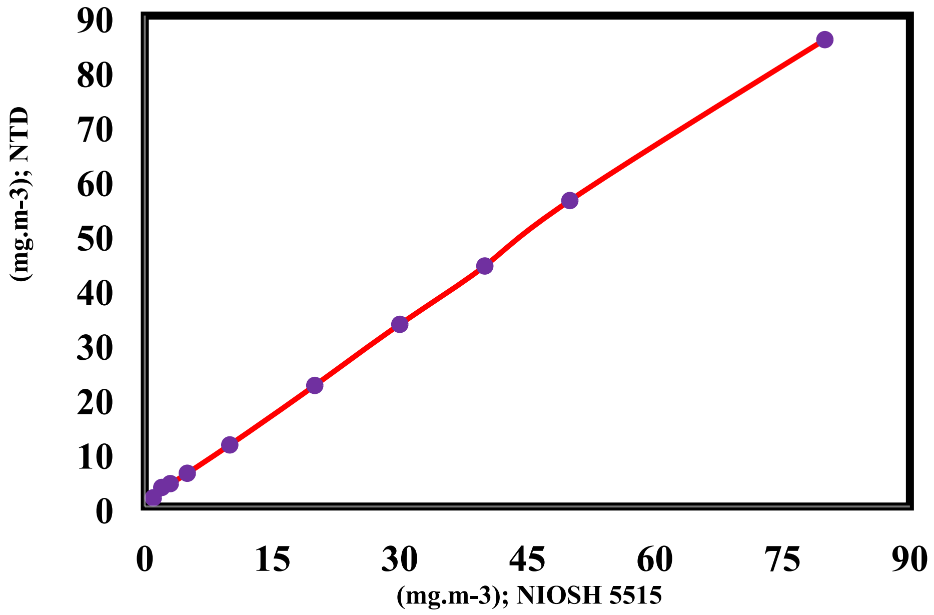

Figure S2. Comparison of the result between the NTD packed with PANI@HA and XAD-2 sorbent tube for the sampling of the benzo[a]pyrene
